# Adaptive ISAR Imaging of Maneuvering Targets Based on a Modified Fourier Transform

**DOI:** 10.3390/s18051370

**Published:** 2018-04-27

**Authors:** Binbin Wang, Shiyou Xu, Wenzhen Wu, Pengjiang Hu, Zengping Chen

**Affiliations:** 1 Science and Technology on Automatic Target Recognition Laboratory, National University of Defense Technology, Changsha 410073, China; nudtatrxu@163.com (S.X.); idminghai@163.com (W.W.); pjhu2012@126.com (P.H.); atrchen@sina.com (Z.C.); 2 College of Information Engineering, Shenzhen University, Shenzhen 518060, China

**Keywords:** inverse synthetic aperture radar (ISAR), maneuvering targets imaging, Doppler focus shift, modified Fourier transform, gradient descent optimization

## Abstract

Focusing on the inverse synthetic aperture radar (ISAR) imaging of maneuvering targets, this paper presents a new imaging method which works well when the target’s maneuvering is not too severe. After translational motion compensation, we describe the equivalent rotation of maneuvering targets by two variables—the relative chirp rate of the linear frequency modulated (LFM) signal and the Doppler focus shift. The first variable indicates the target’s motion status, and the second one represents the possible residual error of the translational motion compensation. With them, a modified Fourier transform matrix is constructed and then used for cross-range compression. Consequently, the imaging of maneuvering is converted into a two-dimensional parameter optimization problem in which a stable and clear ISAR image is guaranteed. A gradient descent optimization scheme is employed to obtain the accurate relative chirp rate and Doppler focus shift. Moreover, we designed an efficient and robust initialization process for the gradient descent method, thus, the well-focused ISAR images of maneuvering targets can be achieved adaptively. Human intervention is not needed, and it is quite convenient for practical ISAR imaging systems. Compared to precedent imaging methods, the new method achieves better imaging quality under reasonable computational cost. Simulation results are provided to validate the effectiveness and advantages of the proposed method.

## 1. Introduction

Inverse synthetic aperture radar (ISAR) imaging plays important roles in civil and military fields, and has attracted the attention of many researchers in the past decades [1,2,3,4,5,6,7,8]. High-range resolution can be obtained by transmitting a wideband signal, and high cross-range resolution is achieved by the rotation angle of the target relative to the light-of-sight (LOS) of the radar [9]. In ISAR imaging scenarios, the motion of the target can be decomposed into translational motion and rotational motion. Only the rotational motion contributes to imaging and the translational motion induces the range misalignment and phase incoherence [10]. Therefore, the translational motion compensation which consists of range alignment and phase compensation [11,12,13,14] is essential.

After translational motion compensation, the range-Doppler (RD) algorithm [15,16] is usually adopted to produce a 2D ISAR image. The RD algorithm assumes that the scatterer’s Doppler frequency is constant during the time period of aperture synthesis. This is suitable for ISAR imaging of targets with smooth motion. For maneuvering targets, due to the equivalent accelerated rotation of the target, the Doppler frequency of each scatterer is time-varying. As a result, the RD algorithm fails and a blurring problem arises in the ISAR image. In order to solve this problem, the range instantaneous Doppler (RID) algorithm [17,18] is proposed.

The RID algorithm can be divided into two categories. One is based on the time-frequency representations [19,20]. The most commonly-used ones include the short-time Fourier transform (STFT) [21], the Wigner-Ville distribution (WVD) [22], the smoothed pseudo-WVD (SPWVD) [23], the reassigned smoothed pseudo-Wigner-Ville distribution (RSPWVD) [24], and so on. STFT is free from the cross-term interference but its resolution is poor. The others belong to the bilinear transform and suffer from the tradeoff between the time frequency concentration and the interference of cross-terms. The second category is based on the parameter estimation of signal model. When the maneuvering of the target is not too severe, the target’s motion could be regarded as a uniform accelerated rotation after translational motion compensation. Additionally, each scatterer brings about a linear frequency modulated (LFM) signal [25,26]. The parameters of the LFM signal, namely the centroid frequency and chirp rate, can be estimated by many effective methods, such as the Radon-Wigner transform (RWT) [27], the fractional Fourier transform (FrFT) [28], etc. However, these methods require estimating the parameters of each LFM component, which are computationally inefficient and inaccurate. Additionally, these RID methods do not consider the possible residual error of translational motion compensation.

In this paper, we study the ISAR imaging of targets with not too severe maneuvering motion, and propose an adaptive imaging method based on modified Fourier transform. After translational motion compensation, the maneuvering motion can be regarded as uniform accelerated rotation and can be described by the rotational angular velocity and acceleration. The jerk of the target is very small and can be neglected. For targets with rigid body, the relative chirp rate of LFM signal is the same for all scatterers and only depends on target’s motion status. Additionally, considering the possible residual error of translational motion compensation, a new variable called Doppler focus shift is defined to describe the shift of the equivalent rotation axis in the azimuth direction. With the relative chirp rate and the Doppler focus shift, both the quadratic phase terms of LFM signals and the rotation axis shift could be compensated in the modified Fourier transform matrix. Then the blurring in ISAR images is effectively eliminated. On this basic, ISAR imaging of maneuvering targets is converted into a parameter optimization problem. The optimal relative chirp rate and Doppler focus shift are obtained through a two-dimensional gradient descent method according to the optimal imaging quality. Moreover, we design an efficient and robust initialization process for the gradient descent method, thus, optimal imaging result could be obtained adaptively without human intervention. This is very helpful and convenient for practical ISAR imaging system. Except for the advantages of clear imaging result, robustness, and automation, the proposed method does not require heavy computation.

The rest of the paper is organized as follows: In Section 2, the ISAR imaging geometry and signal model are presented; the principles of the newly-proposed method are elaborated in Section 3; experimental results based on simulation are provided in Section 4; and, finally, Section 5 draws the conclusions of this paper.

## 2. Imaging Model of the Maneuvering Target

**Figure 1:**
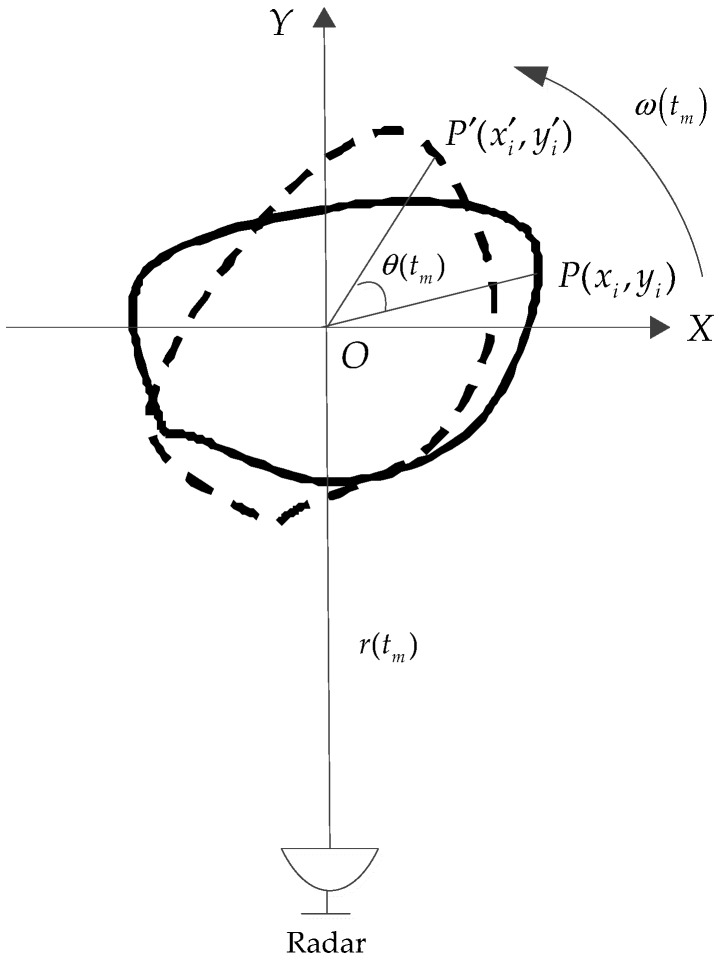
The geometry of ISAR imaging.

The ISAR imaging geometry is shown in Figure 1. *O*-*XY* defines a Cartesian coordinate system, where the origin *O* is located at the rotating axis of the target. The radar is located on the target plane along the *Y*-axis. Assuming that $P(x_{i},y_{i})$ is a scatterer on the target, its range to the radar can be expressed by:

(1) $$R_{i}\left(t_{m}\right)=r(t_{m})+x_{i}\sin\theta (t_{m})+y_{i}\cos\theta (t_{m})$$

where $t_{m}$ is the slow time, $r(t_{m})$ is the distance between the radar and the geometric center of the target, and $\theta (t_{m})$ is the rotated angle of the target relative to the LOS.

Supposing the transmitted LFM signal is:

(2) $$s(\hat{t},t_{m})=rect\left(\frac{\hat{t}}{T_{p}}\right)\exp\left\{j2\pi \left(f_{c}t+\frac{1}{2}\gamma {\hat{t}}^{2}\right)\right\}$$

where $\hat{t}$ is fast time, $\mathrm{rect}(x)=\left\{ \begin{array}{ll} 1 & \left|x\right|\leq 0.5\\ 0 & \left|x\right|>0.5 \end{array}\right.$, $T_{p}$ is the pulse width, $f_{c}$ is the carrier frequency, and γ is the chirp rate. The echo is received through wideband direct sampling, and then matched filtering containing high speed movement compensation [29] is adopted for pulse compression.

After matched filtering, the obtained range profile is expressed as:

(3) $$\begin{array}{c} s\left(\hat{t},t_{m}\right)=\sum_{i=1}^{Q}\sigma_{i}T_{p}\sin c\left\{B\left(\hat{t}-\frac{2\left(r(t_{m})+x_{i}\sin\theta (t_{m})+y_{i}\cos\theta (t_{m})\right)}{c}\right)\right\}\\ \cdot \exp\left\{-j4\pi f_{c}\cdot \frac{r(t_{m})+x_{i}\sin\theta (t_{m})+y_{i}\cos\theta (t_{m})}{c}\right\} \end{array}$$

where $\sigma_{i}$ is the backward scattering coefficient, Q is the total number of scatterers on the target. $\gamma T_{p}$ is the bandwidth of the emitted LFM signal, and c is the propagation velocity of the electromagnetic wave.

After translation motion compensation, the range profile can be written as:

(4) $$\begin{array}{c} s\left(\hat{t},t_{m}\right)=\sum_{i=1}^{Q}\sigma_{i}T_{p}\sin c\left\{B\left(\hat{t}-\frac{2\left(x_{i}\sin\theta (t_{m})+y_{i}\cos\theta (t_{m})\right)}{c}\right)\right\}\\ \cdot \exp\left\{-\frac{j4\pi }{\lambda }\left(x_{i}\sin\theta \left(t_{m}\right)+y_{i}\cos\theta \left(t_{m}\right)\right)\right\} \end{array}$$

Next, we concentrate on the pulse compression along the cross-range direction, so the sinc function related to fast time is neglected in the following discussion. The range profile can be simplified as:

(5) $$s\left(t_{m}\right)=\sum_{i=1}^{Q}\sigma_{i}T_{p}\exp\left\{-\frac{j4\pi }{\lambda }\left(x_{i}\sin\theta \left(t_{m}\right)+y_{i}\cos\theta \left(t_{m}\right)\right)\right\}$$

For targets with smooth motion, the equivalent angular velocity can be denoted as ω, then $\theta \left(t_{m}\right)=\omega t_{m}$. In general, the rotation angle $\theta \left(t_{m}\right)$ is small during the time interval of aperture synthesis, so we have the following approximations:

(6) $$\begin{array}{l} \sin\theta \left(t_{m}\right)\approx \omega t_{m}\\ \cos\theta \left(t_{m}\right)\approx 1 \end{array}$$

Substituting Equation (6) into Equation (5) yields:

(7) $$s\left(t_{m}\right)=\sum_{i=1}^{Q}\sigma_{i}T_{p}\exp\left\{-\frac{j4\pi }{\lambda }\left(x_{i}\omega t_{m}+y_{i}\right)\right\}$$

Obviously, time dependent phase component in Equation (7) corresponds to the cross-range position of the *i*th scatterer. The ISAR image can be obtained by performing Fourier transform with respect to $t_{m}$. We denote the range profile sequence as G(m,n), where *m* represents the pulse number related to slow time, *n* represents range cell related to fast time. There are M pulses and N range cells in total. We denote the discrete ISAR image as I(m,n) and denote the Fourier transform matrix as *P*. The process of RD algorithm can be expressed as:

(8) I=P⋅G

However, for maneuvering targets, the equivalent angular velocity is time-varying and the rotation angle is $\theta \left(t_{m}\right)=\omega t_{m}+\frac{1}{2}\alpha t_{m}^{2}$, where α is the equivalent rotational acceleration. In this case, Equation (7) turns into:

(9) $$\begin{array}{cc} s\left(t_{m}\right) & =\sum_{i=1}^{Q}\sigma_{i}T_{p}\exp\left\{-\frac{j4\pi }{\lambda }\left(x_{i}\omega t_{m}+\frac{1}{2}x_{i}\alpha t_{m}^{2}+y_{i}\right)\right\}\\ & =\sum_{i=1}^{Q}\sigma_{i}T_{p}\exp\left(-j2\pi \left(f_{i}t_{m}+\frac{1}{2}k_{i}t_{m}^{2}\right)\right)\exp\left(-\frac{j4\pi y_{i}}{\lambda }\right) \end{array}$$

where $f_{i}=\frac{2x_{i}\omega }{\lambda }$, $k_{i}=\frac{2x_{i}\alpha }{\lambda }$. Equation (9) indicates that the cross-range signal is a multicomponent LFM signal. The centroid frequency $f_{i}$ and the chirp rate $k_{i}$ depend on the scatterer’s azimuth coordinate and rotation parameters. The quadratic phase term of LFM signal leads to the blurring of ISAR image, thus, it must be compensated. We aim to analyze, construct, and compensate the quadratic phase terms in the following section.

## 3. Adaptive Imaging Method Based on a Modified Fourier Transform

### 3.1. Compensation of Quadratic Phase Terms

The mathematic definition of relative chirp rate is:

(10) $$K_{\alpha \omega }=\frac{k_{i}}{f_{i}}=\frac{\alpha }{\omega }$$

Obviously, $K_{\alpha \omega }$ depends on target’s rotation status and it is the same for all scatterers. According to Equation (9), it could be seen that apart from the slow time, the quadratic phase terms consists two variables: scatterer’s azimuth coordinate $x_{i}$ and equivalent angular acceleration α. However, it is difficult to obtain $x_{i}$ when the ISAR image is blurring. Additionally, it is difficult to estimate the precise equivalent angular acceleration. Nevertheless, it is worth noting that the angular acceleration α is identical for all scatterers and azimuth coordinates are finite values. In the discrete ISAR image, the azimuth coordinate of a scatterer could be expressed as $x_{i}\approx m^{\prime }\rho_{a}$, where $m^{\prime }$ represents image’s Doppler cell index in which the scatterer is located, and $\rho_{a}$ is the azimuth resolution. Apparently, $\rho_{a}=\frac{\lambda }{2\cdot \Delta \theta }$ is identical for all scatterers, where Δθ is the rotated angle of target during the time interval of aperture synthesis. The compensation component for quadratic terms in Equation (9) could be approximated as:

(11) $$\begin{array}{cc} \varphi_{m^{\prime }}\left(t_{m}\right) & \approx \exp\left(\frac{j4\pi }{\lambda }\cdot \frac{1}{2}m^{\prime }\rho_{a}\alpha t_{m}^{2}\right)\\ & =\exp\left(\frac{j2\pi f_{c}}{c}\frac{\lambda }{2\Delta \theta }m^{\prime }\alpha t_{m}^{2}\right)\\ & =\exp\left[\frac{j2\pi f_{c}}{c}\frac{\lambda }{2\left(MT+0.5K_{\alpha \omega }{\left(MT\right)}^{2}\right)}K_{\alpha \omega }m^{\prime }t_{m}^{2}\right] \end{array}$$

where *T* is the pulse repetition interval. The features of $\varphi_{m^{\prime }}\left(t_{m}\right)$ could be listed as follows:

(1) For all scatterers on the target, the relative chirp rate $K_{\alpha \omega }$ is identical.
(2) For scatterers located in the same Doppler cell, their compensation components for the quadratic phase terms are approximately identical.
(3) The values of $m^{\prime }$ represent the discrete azimuth positions of scatterers, and they are related to different Doppler cells in ISAR image.

Each Doppler cell in ISAR image is related to a row vector in Fourier transform matrix *P*. After getting the exact value of $\varphi_{m^{\prime }}\left(t_{m}\right)$, it is feasible to compensate the quadratic phase terms in matrix *P*. We denote $W=\exp\left(-j\frac{2\pi }{M}\right)$, the row vector of matrix *P* can be expressed as:

(12) $$row\_vector=\left[\begin{array}{cccc} W^{\left(-M/2\right)m^{\prime }} & W^{\left(-M/2+1\right)m^{\prime }} & \cdots & W^{\left(M/2-1\right)m^{\prime }} \end{array}\right]$$

From the first row to the *M*th row, the value of $m^{\prime }$ is $m^{\prime }=-M/2,-M/2+1,\ldots ,M/2-1$. The row vector can be simplified as:

(13) $$row\_vector=\left[W^{mm^{\prime }}\right]$$

where  m=−M/2,−M/2+1,…,M/2−1 represents discrete slow time. The compensation components for row vectors of matrix *P* can be expressed as:

(14) $$\begin{array}{cc} \varphi_{m^{\prime }}\left(m\right) & =\exp\left[\frac{j2\pi f_{c}}{c}\frac{\lambda }{2\left(\omega MT+0.5\alpha {\left(MT\right)}^{2}\right)}\alpha m^{\prime }m^{2}T^{2}\right]\\ & =W^{-m^{\prime }m^{2}\frac{K_{\alpha \omega }T}{2\left(1+0.5K_{\alpha \omega }MT\right)}} \end{array}$$

We denote the Fourier transform matrix containing quadratic phase compensation as $P^{\prime }$ and name it the modified Fourier transform matrix. The imaging formula for maneuvering targets could be written as:

(15) $$I=P^{\prime }\cdot G$$

It should be noted that for targets with smooth motion, the relative chirp rate $K_{\alpha \omega }$ is zero, and then $P^{\prime }$ degrades into conventional Fourier transform matrix *P*. That is to say, our proposed algorithm is a generalized version of the classical RD algorithm.

### 3.2. Doppler Focus Shift

The analysis above builds upon an implicit assumption that the coefficients of quadratic phase terms are proportional to the scatterer’s azimuth coordinate and Doppler frequency. This assumption requires that the equivalent rotation axis is located at the center of the azimuth direction, and it is reasonable when there is no error in the translational motion compensation process. However, when the entropy-minimization-based autofocusing technique [30] is adopted for translational phase compensation, the equivalent rotation axis may shift due to the asymmetric pose of the target.

The cross-range cell in which the equivalent rotation axis locates is denoted as Doppler focus. The shifted cell number of Doppler focus caused by an inaccurate translational motion compensation is denoted as Doppler focus shift. When the Doppler focus shift is considered, the compensation term in Equation (14) turns into:

(16) $$\phi_{m^{\prime }}\left(m\right)=W^{-\left(m^{\prime }+m_{shift}\right)m^{2}\frac{K_{\alpha \omega }T}{2\left(1+0.5K_{\alpha \omega }MT\right)}}$$

where $m_{shift}$ is the Doppler focus shift. The cross-range cell in which the Doppler focus locates satisfies $m^{\prime }+m_{shift}=0$, thus, the corresponding quadratic phase terms is zero and the blurring does not exist. On this basic, the value of the Doppler focus shift can roughly be estimated by direct observation of the target’s RD image. Take the offset of the clearest cross-range cell to the center of the cross-range cells as the coarse value of the Doppler focus shift. The accurate value could be obtained by a parameter search. It can be seen from Equation (16) that the relative chirp rate $K_{\alpha \omega }$ and the Doppler focus shift $m_{shift}$ couple with each other, so it is necessary to obtain their exact values by means of a two-dimensional parameter search.

### 3.3. Parameter Search by the Two-Dimensional Gradient Descent Optimization Scheme

Equation (9) indicates that the relative chirp rate can be got by estimating the centroid frequency and the chirp rate of LFM component in the cross-range signal. Several methods, such as FrFT, Lv’s distribution (LVD) [31] work well for parameter estimation of the LFM signal. However, because of the interference of noise and other adverse factors, the relative chirp rate obtained by parameter estimation is only a coarse value. Meanwhile, the value of Doppler focus shift obtained through direct observation is not accurate enough, which also degrades the imaging result. To solve this problem, we adopt parameter searching scheme to get optimal relative chirp rate and Doppler focus shift. Firstly, set the searching ranges of the relative chirp rate and Doppler focus shift. Secondly, set the searching step lengths according to accuracy demands. Thirdly, we carry out the modified Fourier transform and compute the image entropy. The image entropy function is adopted as a criterion to measure the quality of the ISAR image [32]. The mathematical definition of the ISAR image entropy can be written as [7,33]:

(17) $$H=-\sum_{m=1}^{M}\sum_{n=1}^{N}p(m,n)\cdot \ln\left\{p(m,n)\right\},\;where\;p(m,n)=\frac{\left|I(m,n)\right|}{\sum_{m=1}^{M}\sum_{n=1}^{N}\left|I(m,n)\right|}$$

A well-focused image results in small entropy. The accurate relative chirp rate and Doppler focus shift can be obtained via minimizing the entropy of the ISAR image.

However, the parameter searching process above is a brute force solution. Though feasible, it is costly in computation. In general, the change of the image entropy is smooth, the minimum entropy corresponding to the optimal ISAR image is unique, thus, the parameter searching process is a convex optimization problem. The gradient descent method is promising in obtaining the global optimization of the convex object function [34,35]. The basics of the gradient descent method make use of the property that the function value reduces fastest in the negative gradient direction. After several iterations, the iteration point can approach the minimum of the object function. Through the two-dimensional gradient descent process, minimum entropy of the ISAR images along with the accurate relative chirp rate and Doppler focus shift can be obtained. The searching process for $K_{\alpha \omega }$ and $m_{shift}$ in this paper is summarized as follows:

Step 1: $m_{shift}$ is held zero. The relative chirp rate is set $K_{\alpha \omega }=10^{x}$, where x=−4,−3,−2,−1,0,1,2,3,4. Then carry out the modified Fourier transform and compute the corresponding image entropy.

Step 2: Determine the minimum image entropy and the corresponding relative chirp rate, denoted as $10^{x}{}^{*}$. Set $10^{x*}$ and $10^{x*-1}$ as the initial value and step length of $K_{\alpha \omega }$ respectively.

Step 3: Let $K_{\alpha \omega }=10^{x*}$, compute the image entropy under Doppler focus shift $m_{shift}=\pm 10^{y}$, where y=0,1,2.

Step 4: Search for the minimum entropy and the corresponding Doppler focus shift $10^{y*}$. Set $10^{y*}$ and $10^{y*-1}$ as the initial value and step length of $m_{shift}$ respectively.

Step 5: Carry out the two-dimensional gradient descent process based on the initial value and step length of $K_{\alpha \omega }$ and $m_{shift}$. In each iteration, the partial gradient $\frac{\partial E\left(K_{\alpha \omega },m_{shift}\right)}{\partial K_{\alpha \omega }}$ and $\frac{\partial E\left(K_{\alpha \omega },m_{shift}\right)}{\partial m_{shift}}$ are calculated to update $K_{\alpha \omega }$ and $m_{shift}$.

Step 6: When the image entropy change between two adjacent iterations is smaller than preset threshold, the iteration stops. The relative chirp rate and Doppler focus shift in last iteration are output as optimal values.

Steps 1–4 is the initialization process of the two-dimensional gradient descent method. This process not only considers the wide value ranges of $K_{\alpha \omega }$ and $m_{shift}$, but also obtains the coarse initial values for gradient descent optimization quickly. Compared with parameter estimation methods, this initialization process is not accurate, but it is fast and efficient. Setting the step length as 1/10 of the initial value ensures both the convergence and the convergence rate of the optimization process. With this efficient and robust initialization process, the optimal relative chirp rate and Doppler focus shift can be obtained adaptively, and then a well-focused ISAR image is obtained.

In our new method, the modified Fourier transform, instead of the conventional Fourier transform, is used to perform the azimuth compression. When the relative chirp rate and Doppler focus shift are zero, the modified Fourier transform degrades to the conventional Fourier transform. Our new method unifies the imaging problem of smooth targets and maneuvering targets into the same imaging model. Meanwhile, with the efficient initialization process and two-dimensional gradient descent optimization, our method can achieve the well-focused images of maneuvering targets adaptively.

**Figure 2:**
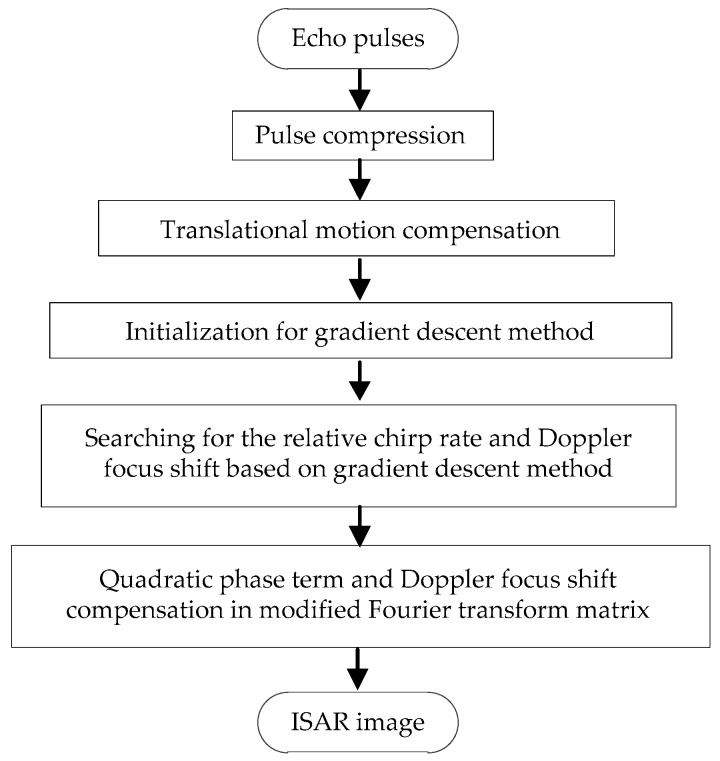
Flowchart of the new ISAR imaging method.

To summarize, the flowchart of the proposed ISAR imaging method is shown in Figure 2.

## 4. Simulation Results

In this section, the new imaging method is verified using simulated data. Imaging results of precedent methods are provided as a comparison.

### 4.1. Results of a Simulated Airplane

**Figure 3:**
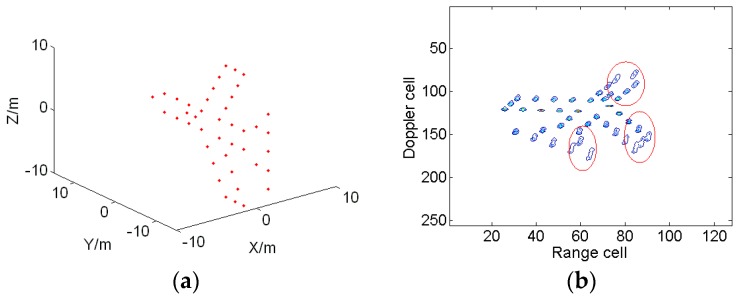
Target model and ISAR imaging result. (**a**) The target model; and (**b**) the ISAR image based on the RD algorithm.

The parameters of the radar are given in Table 1. A plane model containing 42 scatterers, as shown in Figure 3a, is assumed to be the target. The backward scattering coefficients of all scatterers are set as 1. The target’s position in the radar coordinate system is (3000, 3000, 7000) m, the target’s speed is *v* = (225, 300, 0) m/s, and the target’s acceleration is *a* = (84, 112, 0) m/s^2^. Modified envelope correlation [14] and the entropy-minimization-based autofocusing technique [30] are applied to complete the range alignment and phase compensation, respectively. The ISAR image obtained by the conventional RD algorithm is illustrated in Figure 3b. It is obvious that the image is smeared due to the maneuvering of the target.

**Figure 4:**
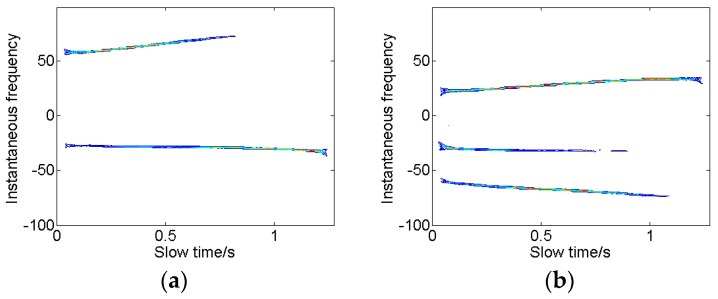
Time-frequency representation of the cross-range signal in the selected range bin. (**a**) The 65th range bin; and (**b**) the 76th range bin.

Then, we compute the time-frequency representations of the cross-range signals using RSPWVD, as shown in Figure 4. Here, the cross-range signals in the 65th and 76th range cells are selected. Two and three scatterers could be identified in these two range cells, respectively. In Figure 4a, the second line is almost parallel to the slow time axis, but it does not locate in the center of Doppler cell, which indicates the Doppler focus has shifted. The Doppler frequency in Figure 4 varies uniformly with the slow time axis, which indicates that the cross-range signals satisfy multicomponent LFM model.

**Figure 5:**
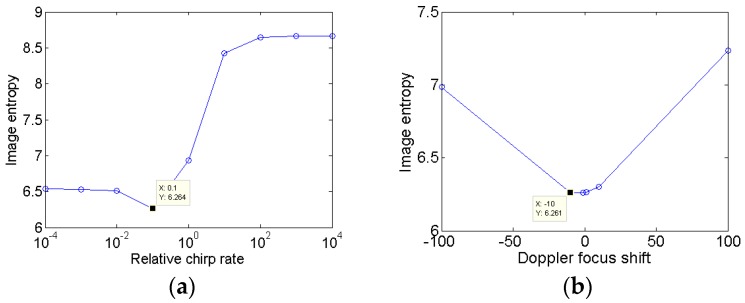
Initialization for gradient descent method. (**a**) The image entropy versus the relative chirp rate; and (**b**) the image entropy versus the Doppler focus shift.

In the following, the proposed algorithm is used to generate the ISAR images. Figure 5 illustrates the image entropy versus the relative chirp rate and Doppler focus shift. It could be seen that the relative chirp rate corresponding to minimum entropy is 0.1, and the coarse estimation of the Doppler focus shift is −10. Thus, the search step lengths for and are set as 0.01 and −1, respectively. The termination condition is set that the image entropy change between adjacent iterations is smaller than 0.0001.

**Figure 6:**
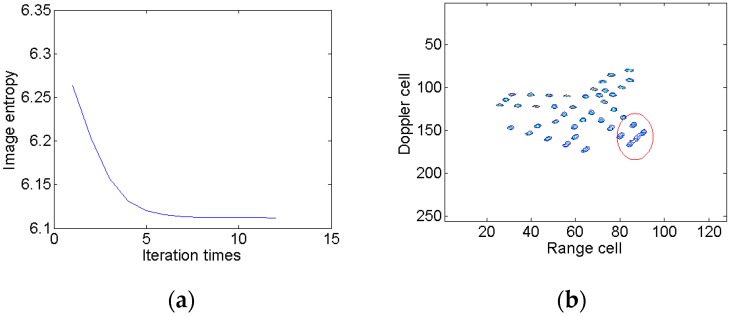
Searching for the optimal relative chirp rate. (**a**) Image entropy versus the iteration times; and (**b**) the optimal ISAR image result.

**Figure 7:**
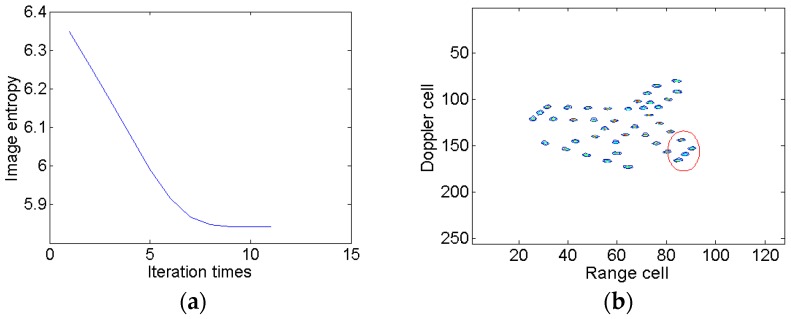
Searching for the optimal relative chirp rate and Doppler focus shift. (**a**) Image entropy versus the iteration times; and (**b**) the optimal ISAR image result.

To demonstrate the coupling between $K_{\alpha \omega }$ and $m_{shift}$, two schemes are carried out in a modified Fourier transform. At first, only the relative chirp rate is considered. The image entropy versus the iteration times is shown in Figure 6a, from which we can see that the image entropy decreases gradually. After 12 iterations, the termination condition is met and the optimal relative chirp rate is 0.20. The optimal image is illustrated in Figure 6b, which indicates that the blurring problem is effectively alleviated compared to Figure 3b. However, the scatterers in the red ellipse are still slightly blurred. Then, we take the Doppler focus shift into consideration. The change of image entropy is shown in Figure 7a. After 11 iterations, the iteration comes to an end. The optimal relative chirp rate and Doppler focus shift are 0.28 and −9.99, respectively. The final image is shown in Figure 7b. It is seen that the image is well focused in both the range and azimuth directions and the blurring in Figure 6b is effectively eliminated.

**Figure 8:**
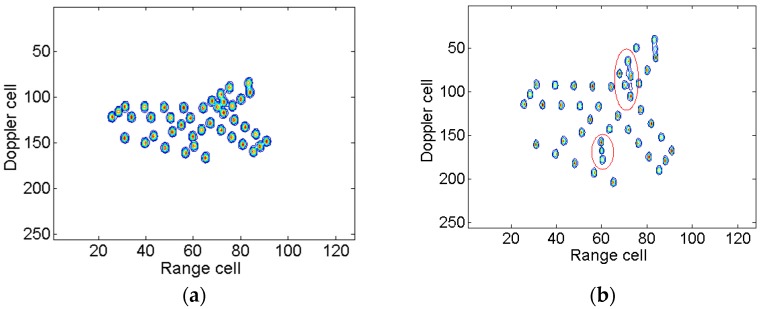
RID images. (**a**) RID image based on STFT at time 0.64 s; and (**b**) RID image based on SPWVD at time 0.64 s.

Figure 8 shows the RID images obtained by taking the slices along the cross-range frequency domain. Additionally, the images are utilized to compare with that obtained through the proposed ISAR imaging method. The RID image produced by STFT algorithm is shown in Figure 8a. Due to a time-limited window function, the frequency resolution is low and the image is not well-focused. Figure 8b is the RID image obtained by SPWVD, in which spurious scatterers exist, especially in the region denoted by red the ellipse.

Image contrast is an alternative criterion to evaluate the image quality. Additionally, the mathematical definition of image contrast [7] is:

(18) $$C=\frac{\sqrt{E\left\{{\left[\left|I(m,n)\right|-E\left(\left|I(m,n)\right|\right)\right]}^{2}\right\}}}{E\left(\left|I(m,n)\right|\right)}$$

where E(·) represents the average operation. The well-focused image results in large contrast.

The entropies and contrasts of the ISAR images based on different methods are listed in Table 2. We can see that the image entropy in Figure 6b is smaller than those obtained by the other three conventional methods, which indicates that the proposed method achieves better image quality than the RID algorithm even if Doppler focus shift is not considered. The ISAR image with minimum entropy is Figure 7b. However, the image with maximum contrast is Figure 8b. Comparing Figure 7b with Figure 8b, both of them are well-focused. However, spurious scatterers exist, as indicated by the red ellipses in Figure 8b. It is found that neither entropy nor contrast are perfect to evaluate the image quality. The two functions will conflict with each other in some special circumstances. This leads to confusion and inconvenience in practice sometimes. According to our practical experience the entropy function is much more reliable than contrast. Hence, the image entropy is adopted as the criterion in the previous discussion.

**Figure 9:**
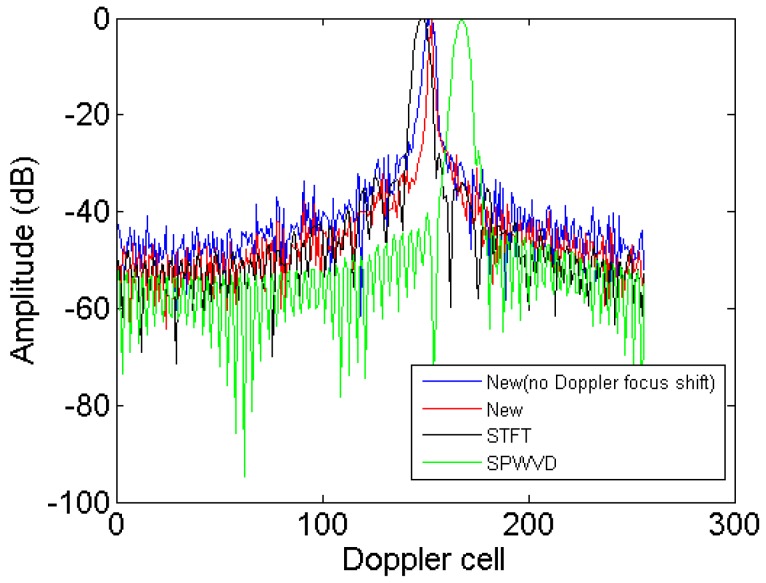
Azimuth profiles of the 91st range cell.

To more clearly compare the image resolution obtained by the new method, STFT and SPWVD, the azimuth profiles in the 91st range cell obtained by these algorithms are given in Figure 9. It is seen that the new method achieves the highest resolution and energy concentration, which further validates its superior performance on resolution improvement.

Simulation times of these methods are listed in Table 3, which are obtained through MATLAB R2014a software on a Lenovo laptop computer with an Intel(R) Core(TM) i5-6300 HQ CPU and 8 GB RAM. From the table, the simulation time of the new method is higher than that of RD and STFT, but lower than SPWVD. Hence, the effectiveness and efficiency of the new imaging method are verified.

### 4.2. Results of the Boeing B727

**Figure 10:**
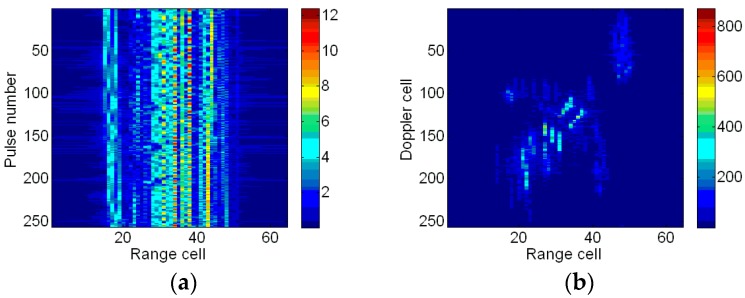
HRRPs and ISAR imaging result. (**a**) HRRPs; and (**b**) ISAR image based on the RD algorithm.

Now, the proposed method is tested on the simulated data of a Boeing B727 airplane provided by the U.S. Naval Research Laboratory [36]. The data contains 256 successive pulses. The carrier frequency is 9 GHz, the bandwidth is 150 MHz, and the PRF is 20 KHz. Translational motion compensation has been accomplished. The high-resolution range profiles (HRRPs) are shown in Figure 10a and the corresponding ISAR image based on the RD algorithm is given in Figure 10b. Due to the maneuvering of the target, the ISAR image is blurred in the cross-range direction.

**Figure 11:**
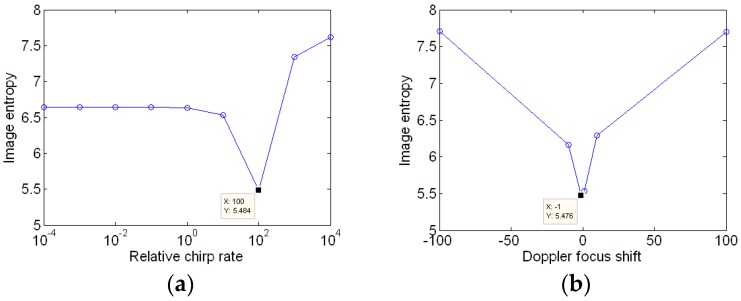
Initialization for gradient descent method. (**a**) Image entropy versus the relative chirp rate; and (**b**) image entropy versus the Doppler focus shift.

Next, the new method is applied to obtain a well-focused image. Simulation results are shown in Figure 11, which indicate that the coarse value of relative chirp rate and Doppler focus shift are 100 and −1, respectively. Then the search step for accurate relative chirp rate and Doppler focus shift are set as 10 and −0.1, respectively. The termination condition is the same as the former experiment.

**Figure 12:**
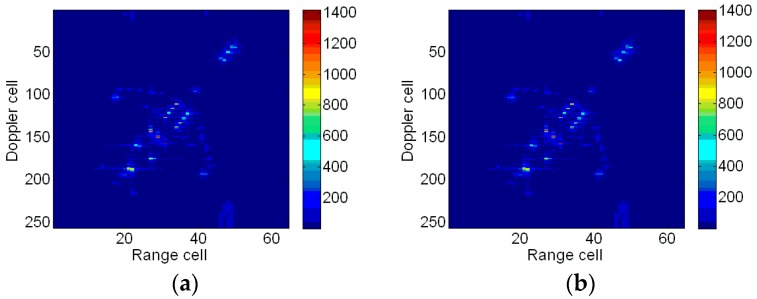
ISAR imaging results. (**a**) ISAR image with optimal relative chirp rate; and (**b**) ISAR image with an optimal relative chirp rate and Doppler focus shift.

Firstly, the Doppler focus shift is not considered, only the relative chirp rate is considered. Simulation results indicate that the optimal relative chirp rate is 107.13. The optimal imaging result is shown in Figure 12a, which is much clearer than that of Figure 10b. Secondly, both the relative chirp rate and Doppler focus shift are considered. After 179 iterations, the termination condition is met. Simulation results indicate that the optimal relative chirp rate and Doppler focus shift are 106.57 and −0.52, respectively. The final imaging result is shown in Figure 12b. Comparing Figure 12b with Figure 12a, the ISAR image is almost the same visually. It could be concluded that the Doppler focus shift is small, so it can be neglected.

**Figure 13:**
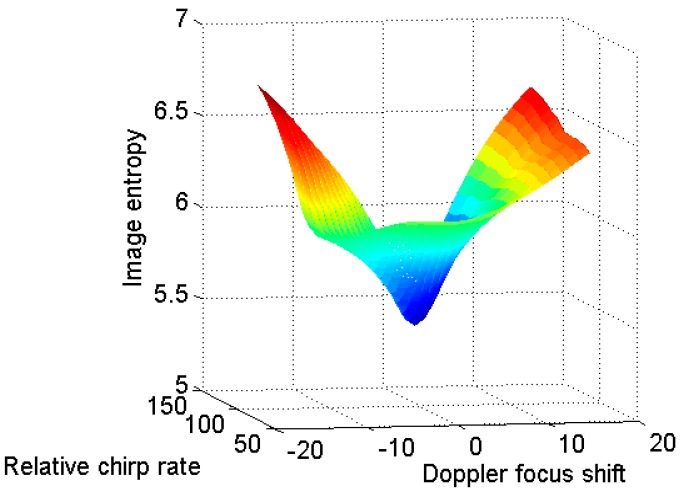
Image entropy versus the relative chirp rate and Doppler focus shift.

The image entropy versus the relative chirp rate and Doppler focus shift is shown in Figure 13. It is seen that the change of image entropy *E*(*K_αω_*,*m_shift_*) is a convex function. The point corresponding to the minimum image entropy is unique. Therefore, the parameters $K_{\alpha \omega }$ and $m_{shift}$ can be obtained by the two-dimensional gradient descent method. The new method is based on the fact that the change of entropy with relative chirp rate and Doppler focus shift is a convex function, and then introducing the two-dimensional gradient descent method to achieve the adaptive feature.

**Figure 14:**
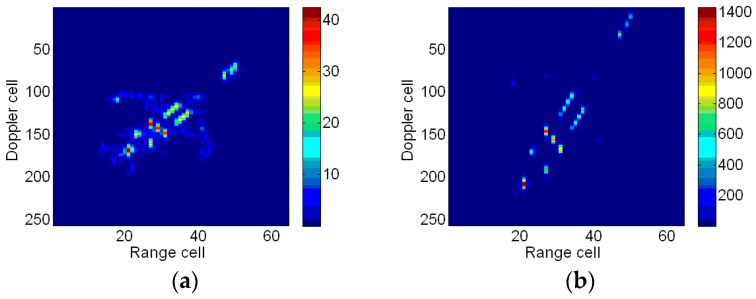
RID images. (**a**) RID image based on STFT at time 0.0014s; and (**b**) RID image based on SPWVD at time 0.0014 s.

Figure 14a shows the RID image produced by STFT algorithm. Suffering from low resolutions, the RID image obtained by STFT is not clear. Figure 14b shows the image produced by SPWVD algorithm. In Figure 14b, some scatterers are invisible, which leads to the loss of the target shape information. It is difficult to distinguish an airplane from the image.

In Table 4, the entropies and contrasts of the above Boeing B727 images are given. It can be seen that the entropies of Figure 12a,b are smaller than that of Figure 10b and Figure 14a, which demonstrate the effectiveness of proposed imaging method. Although the image entropy of Figure 14b is smaller than the new method, the wings and tail of the airplane are invisible in the Figure 14b. It is worth mentioning that the image entropy and contrast conflict with each other in this circumstance.

**Figure 15:**
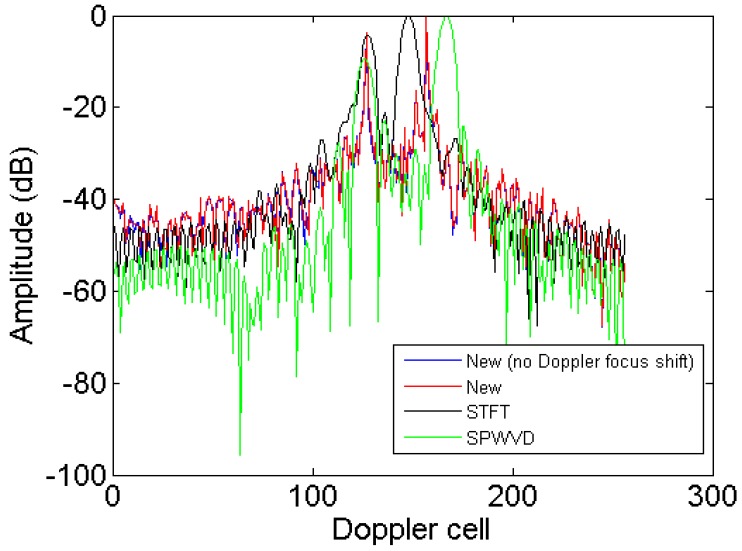
Azimuth profiles of 34th range cell.

To more clearly compare the image resolution obtained by the new method, STFT and SPWVD, the azimuth profiles in the 34th range cell obtained by these algorithms are given in Figure 15. It is seen that the new method achieves the highest energy concentration, which further validates its superior performance on resolution improvement.

## 5. Conclusions

In this paper, an adaptive ISAR imaging method based on a modified Fourier transform is proposed for targets with not too severe maneuvering. In our new method, the relative chirp rate and Doppler focus shift are used to describe the target’s motion after translational motion compensation. The relative chirp rate depends on the maneuvering status of the target. Additionally, the Doppler focus shift presents the possible residual error of translational motion compensation. With them, compensations are carried out in a modified Fourier transform matrix, and the imaging problem is converted into a two-dimensional parameter optimization. Through an efficient and robust initialization, and the consequent two-dimensional gradient descent process, the optimal imaging result could be obtained adaptively. Compared with precedent ISAR imaging methods for maneuvering targets, the new method has the following features: tolerance for translational motion compensation error, clear and stable imaging result, little computational complexity, and excellent convenience for practical application. The results of the simulated data are provided to validate the effectiveness and efficiency of the proposed method.

## Figures and Tables

**Table 1:** Parameters of the radar system.

Parameter	Setting Value	Parameter	Setting Value
Carrier frequency	10 GHz	Bandwidth	500 MHz
Pulse width	50 µs	Complex sampling rate	500 MHz
Pulse repeat frequency	200 Hz	Pulse number	256

**Table 2:** Entropies of ISAR images.

Method	Figure	Entropy	Contrast
RD	Figure 3b	6.75	4.09
New (no Doppler focus shift)	Figure 6b	6.11	4.59
New	Figure 7b	5.84	4.80
STFT	Figure 8a	6.70	4.55
SPWVD	Figure 8b	6.38	5.85

**Table 3:** Computational complexity comparison.

Method	Figure	Simulation Time
RD	Figure 3b	0.09s
New (no Doppler focus shift)	Figure 6b	2.21s
New	Figure 7b	3.62s
STFT	Figure 8a	3.43s
SPWVD	Figure 8b	19.02s

**Table 4:** Entropies of the Boeing B727 images.

Method	Figure	Entropy	Contrast
RD	Figure 10b	6.64	2.40
New (no Doppler focus shift)	Figure 12a	5.43	3.10
New	Figure 12b	5.43	3.10
STFT	Figure 14a	5.80	3.90
SPWVD	Figure 14b	4.75	7.49
